# Hereditary Transmission of Insanity

**Published:** 1847-10

**Authors:** 


5. *Hereditary Transmission of Insanity.*—M. Baillarger, in his "*Statistical researches on the Hereditary Transmission of Insanity,*" arrives at the following conclusions:

1st. The insanity of the mother is more readily transmitted than that of the father.

2d. The mother's insanity is more apt to affect her daughters than her sons: that of the father is more apt to affect the sons.

3d. Sons are not more apt to derive insanity from the mother than from the father; but daughters are most subject to the insanity of the mother.—Translated from Gaz. Medicale de Paris for *Ibid.*

